# Bone-Penetrating TASER Bolt: A Rare Case of Penetrating Injury to the Middle Phalanx of the Little Finger

**DOI:** 10.7759/cureus.19461

**Published:** 2021-11-11

**Authors:** Mohamed Abdelaty, Mahmoud A Kandil, Karl Walsh

**Affiliations:** 1 Plastic and Reconstructive Surgery, Bradford Teaching Hospitals NHS Foundation Trust, Bradford, GBR; 2 Plastic and Reconstructive Surgery, Manchester University NHS Foundation Trust, Manchester, GBR

**Keywords:** finger, upper limb, foreign body, non-lethal weapon, taser

## Abstract

TASER^©^ (Axon Enterprise, Scottsdale, Arizona) is a type of electric gun that delivers a sudden shock to temporarily disable a human subject. It is used by law enforcement, as well as civilians, worldwide. Despite its wide use as a “non-lethal” form of self-defence, it can lead to serious injuries. We are presenting a rare case report of a 28-year-old man who presented to our Plastic Surgery Trauma Service following a penetrating TASER bolt injury to the middle phalanx of his left little finger.

## Introduction

TASER^©^ guns (Axon Enterprise, Scottsdale, Arizona) fire barbed dart-like electrodes attached by wires to penetrate the subject’s skin and deliver an immediate electric shock. This device is believed to deliver electricity potentially as high as 50,000 volts [1-2]. Once the electricity is passed through the electrodes, skeletal muscles contract, stimulating motor neurons until a refractory period is achieved, leaving the affected subject in a state of paralysis. They are becoming increasingly popular among law enforcement forces worldwide as a replacement for firearms [3].

Serious injuries resulting from the direct penetration of the electrodes have been reported in the literature [4-8]. Injury to the fingers has been reported, causing underlying structural damage requiring formal surgical exploration and repair under regional anaesthesia [1]. The purpose of this case report is to highlight a serious outcome from using this product, as the bolt had penetrated through multiple tissue planes including bone. The patient underwent exploration under general anaesthetic to know the extent of damage, both for medical and legal purposes.

## Case presentation

An otherwise fit and healthy 28-year-old man with no previous hand injuries presented to the accident and emergency department after midnight with a retained bolt in his little finger. While resisting police arrest, the police had to use TASER guns to detain him. The patient was awake with no other significant injuries and reported that he was hit with bolts from a distance of approximately two meters. Police officers were able to remove all bolts from his forearms and thighs except for this one in his left little finger, which was fixed to the middle phalanx. After primary and secondary surveys where no other injuries were noted, the patient was referred to the Plastic Surgery Trauma Service. Clinical examination revealed that the bolt entered the dorsum of the middle phalanx with no exit wound. Capillary refill was normal at the tip of the little finger, he had normal sensations in both digital nerves distribution, and the flexor and the extensor tendons were intact.

**Figure 1:**
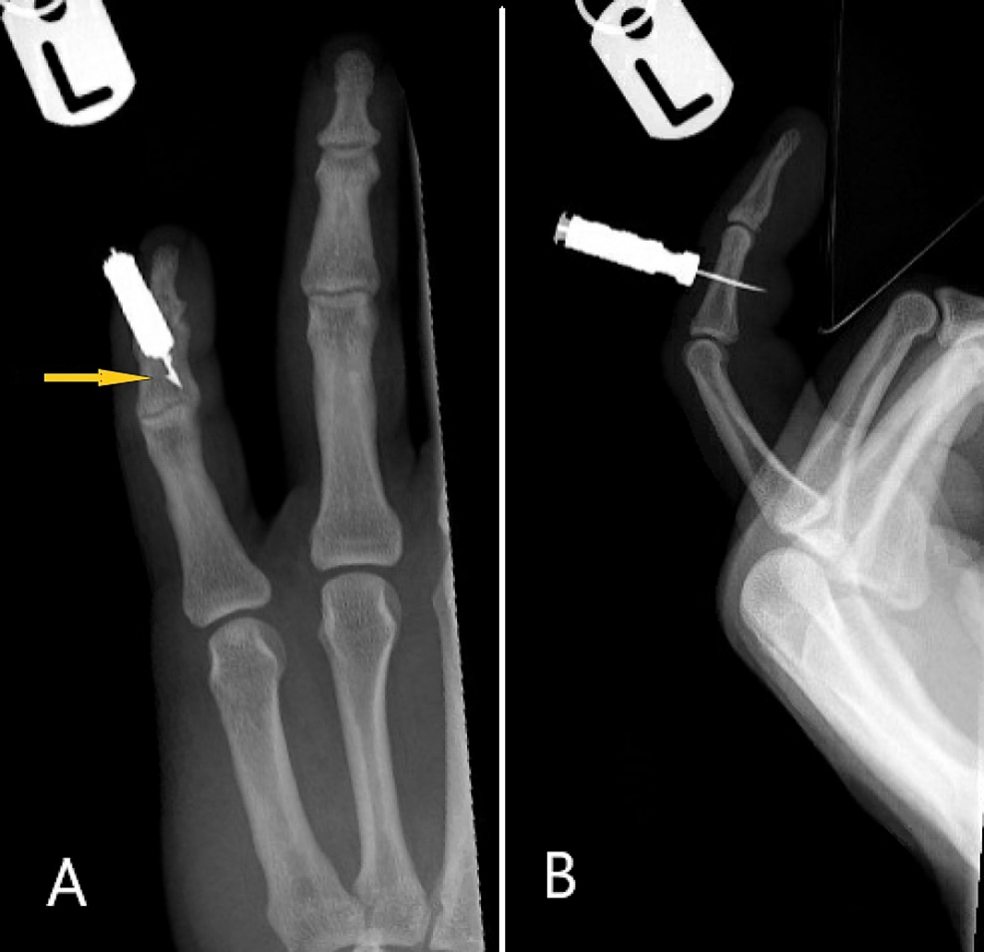
Plain radiographs of the little finger (A) The anteroposterior view shows the TASER bolt with the barbed element (yellow arrow). (B) The lateral view shows the TASER bolt extending through the middle phalanx into the volar soft tissue.

His X-ray (Figure 1) showed the bolt penetrating both the cortices of the middle phalanx from dorsal to volar with the tip ending in volar soft tissues. From a clinical point of view, the main concern was that the deep, pointed part is barbed at right angles, which would suggest difficult retrieval through the same entry point (similar concept to fish hook barbs). The patient refused overnight stay, as he was in police custody and preferred to return to receive his treatment the following day. He was discharged with oral amoxicillin/clavulanate 625 mg three times a day.

Under regional block and arm tourniquet control, the surgeon retrieved the entire bolt through the entry point and confirmed the complete removal using fluoroscopy. The wound was explored dorsally by a lazy S incision, and the extensor tendon was split longitudinally at zone II. A Bruner’s type incision was used to explore the volar aspect. A hematoma was identified within the flexor sheath, but the flexor tendon was found to be intact. The injury was distal to the Flexor Digitorum Superficialis insertion. Both neurovascular bundles were intact. The flexor sheath, as well as the bony tract, were thoroughly washed out with normal saline. No structural repair was required. The patient was later discharged with oral amoxicillin/clavulanate 625 mg for five more days. No further complications were encountered in the immediate post-operative period.

## Discussion

Thomas A Swift's Electric Rifle or TASER^©^ is a type of electric gun that fires two barbed electrodes attached with an isolated wire to deliver an electric shock to a living subject. It was first invented by an American inventor and later became commercially available via TASER international [3]. It comes in different varieties, including military, police, and civilian self-defence grades. Some models have the option of acting as a stun gun when held directly against the subject [1,3].

When fired, the two dart-like electrodes travel at a speed of 50 meters/second up to a range of 10 meters. Once through the person's clothes/skin, they deliver an immediate electric shock of up to 50,000 Volts over five seconds [1]. The physiologic effect of such a shock is stimulation of presynaptic motor neurons, leading to tonic-clonic contractures followed by a refractory period, during which the person affected is paralyzed [3-5].

Even though TASER guns were introduced to law enforcement agencies worldwide as a non-lethal replacement to firearms [6], serious complications have resulted from its use. Apart from the implications of sustaining a mechanical fall with the potential head and bony injury, structural injuries have been reported to the skin such as wounds and burns [1], fractures [7], injury to the eye [4], altered mental status, and even strokes [8].

In our case, the damage was mostly inflicted via the penetrating element of the injury. While upper limb involvement is reported to be present in 7.9% of cases [6], composite finger involvement is rare, with only a few cases published in the literature [1,9]. The injury in our case has involved the extensor tendon, middle phalanx, and flexor sheath, sparing both neurovascular bundles. Although the police officers initially attempted removing the electrode manually, surgical exploration was warranted to identify underlying structural damage and irrigate the affected bone and flexor sheath.

## Conclusions

TASER guns are non-lethal weapons used by law enforcement to control subjects by delivering incapacitating electric shocks. The penetrating element of the gun electrode can result in a severe structural injury. Our case is an example of a finger injury that required surgical exploration under regional anaesthesia with underlying bony and tendon damage. We want to draw the attention of the clinicians to refer cases of phalangeal bolt injuries for the input of a hand surgeon for removal, exploration and structural repair as required.
